# Telomeres and Telomerase in the Radiation Response: Implications for Instability, Reprograming, and Carcinogenesis

**DOI:** 10.3389/fonc.2015.00257

**Published:** 2015-11-24

**Authors:** Brock J. Sishc, Christopher B. Nelson, Miles J. McKenna, Christine L. R. Battaglia, Andrea Herndon, Rupa Idate, Howard L. Liber, Susan M. Bailey

**Affiliations:** ^1^Division of Molecular Radiation Oncology, Department of Radiation Oncology, University of Texas Southwestern Medical Center Dallas, Dallas, TX, USA; ^2^Department of Environmental and Radiological Health Sciences, Colorado State University, Fort Collins, CO, USA

**Keywords:** telomeres, telomerase, ionizing radiation, stem cells, cellular reprograming, instability, carcinogenesis, instability

## Abstract

Telomeres are nucleoprotein complexes comprised of tandem arrays of repetitive DNA sequence that serve to protect chromosomal termini from inappropriate degradation, as well as to prevent these natural DNA ends from being recognized as broken DNA (double-strand breaks) and triggering of inappropriate DNA damage responses. Preservation of telomere length requires telomerase, the specialized reverse transcriptase capable of maintaining telomere length via template-mediated addition of telomeric repeats onto the ends of newly synthesized chromosomes. Loss of either end-capping function or telomere length maintenance has been associated with genomic instability or senescence in a variety of settings; therefore, telomeres and telomerase have well-established connections to cancer and aging. It has long been recognized that oxidative stress promotes shortening of telomeres, and that telomerase activity is a radiation-inducible function. However, the effects of ionizing radiation (IR) exposure on telomeres *per se* are much less well understood and appreciated. To gain a deeper understanding of the roles, telomeres and telomerase play in the response of human cells to IRs of different qualities, we tracked changes in telomeric end-capping function, telomere length, and telomerase activity in panels of mammary epithelial and hematopoietic cell lines exposed to low linear energy transfer (LET) gamma(γ)-rays or high LET, high charge, high energy (HZE) particles, delivered either acutely or at low dose rates. In addition to demonstrating that dysfunctional telomeres contribute to IR-induced mutation frequencies and genome instability, we reveal non-canonical roles for telomerase, in that telomerase activity was required for IR-induced enrichment of mammary epithelial putative stem/progenitor cell populations, a finding also suggestive of cellular reprograming. Taken together, the results reported here establish the critical importance of telomeres and telomerase in the radiation response and, as such, have compelling implications not only for accelerated tumor repopulation following radiation therapy but also for carcinogenic potential following low dose exposures as well, including those of relevance to spaceflight-associated galactic cosmic radiations.

## Introduction

Telomeres, protective features of chromosomal termini composed of tandem arrays of repetitive G/C-rich sequence (5′-TTAGGG-3′ in vertebrates), end in a 3′ single-stranded overhang and are associated with a host of proteins collectively termed “shelterin” (1, 2). Telomere-specific binding proteins include the telomere repeat factors 1 and 2 (TRF1 and TRF2; bind double-stranded telomeric DNA) and protection of telomeres 1 (POT1; binds single-stranded telomeric DNA) (3–5). These end-binding proteins are thought to facilitate invasion of the 3′ single-stranded overhang into the telomeric DNA duplex, forming a lariat-like structure termed a T-loop (6). The T-loop is proposed as an architectural answer to the “end-capping problem,” in that it not only helps protect the end of the chromosome from nucleolytic degradation but also serves to prevent this naturally occurring DNA double-stranded end from being recognized as broken DNA (i.e., a double-strand break; DSB) and initiating inappropriate damage responses (e.g., non-homologous end joining; NHEJ). Due to the semiconservative nature of DNA replication and the requirement for an RNA primer in lagging-strand synthesis, conventional polymerases are unable to replicate to the very end of the chromosome. This so-called “end replication problem” (7, 8) results in progressive erosion of the telomere with each round of cellular division (~30–150 bp per cell division) and is the molecular mechanism underlying the finite replicative lifespan of human somatic cells known as the Hayflick limit (9). Once some stipulated number of telomeres reaches a critically shortened length, a persistent DNA damage response (DDR) is triggered that results in a state of permanent cell cycle arrest known as replicative senescence (10). Direct links between telomere dysfunction – in terms of either significant shortening or compromise of end-capping structure/function – instability and cancer, as well as senescence- and age-related degenerative pathologies (e.g., cardiovascular disease) have been demonstrated (11–13).

Clearly, during carcinogenesis, cells must devise a means of maintaining their telomeres in order to overcome the barrier of senescence and achieve replicative immortality. The overwhelming majority of human cancers (~90%) accomplish this task by the way of reactivation of telomerase (14); the remaining ~10% of cancers maintain telomere length in a telomerase-independent fashion, relying instead on the homologous recombination (HR)-associated alternative lengthening of telomeres (ALT) pathway (15). Telomerase is a specialized reverse transcriptase consisting of a catalytic subunit, telomerase reverse transcriptase (hTERT), which utilizes its telomerase RNA component (hTERC) to synthesize telomeric DNA *de novo* (16, 17). In humans, telomerase activity is transcriptionally repressed in the majority of somatic cells, being expressed at appreciable levels only in adult stem- and germ-line cells (18). It is becoming increasingly appreciated that telomere maintenance and telomerase activity are critical elements of intricate cellular networks that regulate cellular lifespan, genome stability, and carcinogenesis. Indeed, recent studies suggest that telomerase has novel molecular functions well beyond its canonical role in telomere length maintenance, including transcriptional regulation and cellular reprograming, which may well underlie *all* of the hallmarks of cancer (19).

Adult stem cells (SCs), rare subpopulations within tissues that possess extended replicative lifespans by virtue of possessing telomerase activity, are defined by the distinctive properties of self-renewal and the potential to differentiate along various lineages. Deregulation of SC compartments is generally deemed a contributing factor in the development of cancer stem cells (CSCs), which are also referred to as tumor-initiating cells (20). For example, a subpopulation of CSCs (CD44^+^/CD24^low/−^) has been identified in human breast tumors and established breast cancer cell lines that display enhanced tumor-forming capacity in mouse xenograft models (21). Also relevant in this regard, are reports that ionizing radiation (IR) alters the cellular dynamics of tissue and tumor repopulation following exposure and further, that such alteration may be dependent on radiation quality, i.e., linear energy transfer (LET). LET describes the amount of energy an ionizing particle transfers to the material traversed per unit distance and is the predominant factor underlying differences in relative biological effectiveness (RBE) of charged particle vs. photon radiations. For example, high dose per fraction low LET X-ray exposures have been associated with subsequent enrichment of putative CSC populations in a variety of tumor types including breast, colon, lung, prostate, squamous cell carcinoma of the head and neck, and melanoma (22–32). Tang et al. demonstrated that low dose γ-ray and charged particle exposures (Fe and Si ions) in combination with transforming growth factor beta (TGF-β) resulted in increased self-renewal of CK14^+^/CK18^+^SC populations in the humanized mammary fat pads of juvenile mice (33). Such IR-induced SC enrichment has been implicated in radiotherapy failure, accelerated repopulation, and evasion of tumors to CSC targeted therapies (34). Studies increasingly support SCs as critical considerations in the radiation response, whether associated with treatment of cancer (radiotherapy) or exposure of normal tissues (carcinogenesis) as occurs unavoidably in conjunction with radiotherapy and a variety of medical diagnostic procedures, as well as accidentally (e.g., nuclear power plant accidents) and during spaceflight.

It is widely viewed that IR-induced enrichment of CSCs results from mobilization and asymmetric division of existing CSCs, which have been shown to be more radioresistant than their more differentiated non-stem cancer cell (NSCC) counterparts, due not only to their residing in relatively hypoxic niches but also because they possess enhanced DNA repair kinetics, superior endogenous oxidative stress defenses, and slower cell turnover rates (35). Importantly, however, Lagadec et al. have shown that IR-induced enrichment of CD44^+^/CD24^low/−^ and aldehyde dehydrogenase (ALDH) activity high breast CSCs can also result from the reprograming or conversion of NSCCs back into CSCs by inducing expression of transcriptional factors utilized in the generation of induced pluripotent stem cells (iPSCs), e.g., Oct4, Sox2, and Nanog (29). Additional evidence of such “plasticity” was provided by Yang et al., who not only confirmed IR-induced reprograming but also demonstrated that CSC populations maintain an equilibrium within established cell lines, and that the return to equilibrium is facilitated by radiation exposure and TGF-β (23). Therefore, enrichment of CSC populations following radiation exposure may arise either by way of mobilization (i.e., asymmetric division) of existing, radioresistant SC populations in response to injury, or via IR-induced reprograming (i.e., conversion) of NSCCs into CSCs, or perhaps more likely, some combination of the two processes. Implicit in these observations, is the reality that therapeutic strategies seeking to target CSC populations must address both mobilization and reprograming in order to be effective.

Interestingly, the reverse transcriptase component of telomerase (hTERT) has also been implicated as a promoter of “stemness” via interactions with the Wnt/β-catenin signaling and NF-κB inflammation response pathways (36–40), although such findings remain controversial (41, 42). Telomerase activity has also been shown to be radiation-inducible in a variety of tumors and cancer cell lines, including mammary carcinoma, acute myeloid leukemia (AML), colon carcinoma, squamous cell carcinoma of the oral cavity, lymphoma, and nasopharyngeal carcinoma (28, 43–50). Such observations led us to suspect unappreciated correlations between these processes. Furthermore, exposures to high LET, high charge, high energy (HZE) particles, such as those delivered during carbon ion radiotherapy or encountered in the deep space environment, have been shown to invoke very different biological responses than low LET radiations, which may well include IR-induced telomerase activity and subsequent SC enrichment. The effects of IR exposure, particularly radiations of different qualities, on telomere maintenance and/or function are poorly understood [reviewed in Ref. (51)], even though telomeres themselves have been regarded as “hallmarks of radiosensitivity” (52), and recently proposed as informative biomarkers of radiosenstivity for the purposes of personalized medicine (53). Indeed, short telomeres have been shown to enhance IR sensitivity in several settings (54–57), being associated with impaired and/or delayed DSB repair kinetics (54, 58), as well as with persistent chromosomal breaks and cytogenetic profiles characterized by complex aberrations and massive fragmentation (54). However, it is important to note that longer telomeres do not necessarily confer radioresistance. It is also debatable whether such a relationship holds true in telomerase positive cells, as there are reports of no correlation between telomere length and radiosensitivity (59). Other contrasting reports include longer telomeres in irradiated (4 Gy X-rays) vs. unirradiated cells 14 days after exposure (45), as well as significantly shortened telomeres, specifically in the shortest telomere fraction, in the peripheral blood of radiotherapy patients within a relatively short span of time (3 months or less) following treatment for a variety of cancer types (60). Interestingly, low LET X-rays and low energy (high LET) protons have been shown to induce very different telomeric responses, in that telomeres were shortened 96 h post-X-ray exposure and associated with anaphase bridges and dicentrics, while high LET protons evoked telomere lengthening at 24 and 96 h (56).

Considerable controversy and uncertainty surround such results and the processes responsible for them, but accumulating evidence, including much of our own [e.g., Ref. (61–67)], continue to support intimate relationships between functionally intact telomeres and the genomic, cellular, and organismal responses to radiation exposure. Here, we provide new insight into the roles of telomeres and telomerase in the radiation response. Specifically, we investigated the influence of dose, dose rate, and radiation quality on IR-induced changes in telomere function, length, and telomerase activity in panels of cancer and non-cancer mammary epithelial and hematopoietic cells. Depletion of the telomeric end-binding proteins TRF1, TRF2, or POT1 resulted in dysfunctional telomeres that were uncapped as opposed to critically shortened, which (1) increased spontaneous and IR-induced mutation frequencies in a radiation quality-dependent manner, with POT1 depletion being especially effective, and (2) contributed to instability in that they were susceptible to fusion with each other and to IR-induced DSBs, as well as to recombination (telomere sister chromatid exchange; T-SCE). Furthermore, we demonstrate that IR-induced SC enrichment is telomerase dependent, and separate modeling efforts support the necessity of contribution from cellular reprograming for such enrichment (manuscript in preparation, Gao et al.). Better understanding of these fundamental processes involving telomeres and telomerase following IR exposure, particularly of different radiation qualities, is vital, as they play potentially critical roles in accelerated tumor repopulation following radiotherapy, as well as IR-induced carcinogenesis following exposure, including those of relevance to astronauts.

## Materials and Methods

### Cell Culture

The spontaneously immortalized non-tumorigenic human mammary epithelial cell line MCF-10A was purchased from ATCC and was cultured as described previously (68) in 1:1 Dulbecco’s modified essential medium (D-MEM)/Ham’s F12 growth medium (Hyclone) supplemented with 5% fetal bovine serum (FBS), 10 μg/mL insulin (Sigma), 20 ng/mL epidermal growth factor (EGF; Sigma), 0.5 μg/mL hydrocortisone (Sigma), 0.1 μg/mL cholera toxin (Sigma), and 1% GlutaMAX (Gibco, Life Technologies). The human mammary carcinoma cell line MCF-7 (kind gift from L. Chubb, CSU Flint Animal Cancer Center) was grown in D-MEM supplemented with 10% FBS and 1% GlutaMAX. The primary normal mammary epithelial cell line AG11137 (Coriell) was grown in MCDB 170 complete growth medium (US biological) supplemented with 5 μg/mL insulin, 10 ng/mL EGF, 0.5 μg/mL hydrocortisone, 56 μg/mL bovine pituitary extract (Life Technologies), and 1% GlutaMAX.

A human low passage lymphoblastoid cell line (LCL15044, kind gift from A. Sigurdsson, National Institute of Health) was grown in RPMI medium supplemented with 15% FBS and 1% GlutaMAX. The WTK1 human lymphoblastoid cell line was derived from the WI-L2 line (69), and used for mutation analysis, as they are heterozygous at the thymidine kinase (TK) locus; they also have a single amino acid substitution in codon 237 at TP53. WTK1 cells were grown in RPMI medium supplemented with 10% horse serum and 1% GlutaMAX. The human, therapy-induced AML cell line KG1a (kind gift from Michelle LeBeau, University of Chicago) was grown in RPMI media supplemented 20% FBS and 1% GlutaMAX. Normal human peripheral blood mononuclear cells (PBMCs) were collected in accordance with approved IRB protocol [#13-4379H] in 10 mL spray-coated K2EDTA tubes and cultured in PB-MAX karyotyping medium containing the activating mitogen, phytohemagglutinin M (PHA-M) and supplemented with 1% antibiotic/antimycotic (Gibco, Life Technologies).

A variety of control cell lines were included for comparison. Primary BJ-1 normal human foreskin fibroblasts (kind gift from J. Shay, University of Texas Southwestern Medical Center) and hTERT-immortalized BJ-1 (BJ-1-hTERT; kind gift from J. Bedford, Colorado State University) were grown in a 4:1 mixture of D-MEM high glucose medium (Hyclone)/M-199 (Hyclone) supplemented with 10% FBS and 1% GlutaMAX. The human osteosarcoma ALT cell lines U2OS and SAOS2 (kind gift from D. Gustafson CSU Flint Animal Cancer Center) were grown in McCoy’s 5A growth medium (Gibco, Life Technologies) supplemented with 10% FBS and 1% GlutaMAX. The highly telomerase-positive human immortal HeLa cell line was purchased from ATCC and cultured in D-MEM high glucose medium, supplemented with 10% FBS and 1% GlutaMAX. All cells were maintained in a humidified incubator at 37°C in 5% CO_2_ and passaged 1–2 times/week.

### Irradiations and Clonogenic Cell Survival

#### For γ-Ray Exposures

For γ-ray exposures, cells were exposed to various, acute doses of ^137^Cs γ-rays in a Mark I irradiator (J. L. Shepherd) located at Colorado State University. Cells were exposed at a dose rate of 2.5 Gy/min with rotation. For LDR exposures, cells were incubated under a ^137^Cs source to total doses of 1 or 4 Gy γ-rays at dose rates of 4.9 and 3.12 cGy/h. Unirradiated controls were kept in a separate incubator under identical conditions.

#### Exposures to 1 GeV/n ^56^Fe Ions

Exposures to 1 GeV/n ^56^Fe ions (HZE) were delivered at the NASA Space Radiation Laboratory (NSRL), located at Brookhaven National Laboratory (BNL), Upton, NY, USA. Flasks of cells were shipped from Colorado State University in insulated containers at room temperature and were exposed to acute doses of 1 or 2 Gy at a dose rate of ~1 Gy/min. Immediately following exposure, cells were shipped overnight back to Colorado State University for processing and analysis.

#### Clonogenic Cell Survival

MCF-7 and MCF-10A cells were seeded in triplicate 48 h prior to irradiation and allowed to incubate under standard culture conditions to ensure that all cultures were in log phase. Cells were irradiated with an acute dose of 1–10 Gy of ^137^Cs γ-rays. Cells were then allowed to incubate for 16 h overnight to allow for repair to occur. Cells were then trypsinized, counted, and plated at the appropriate density in quadruplicate into 60 mm culture dishes. Cells were allowed to incubate for 10 (MCF-7) or 14 days (MCF-10A). Plates were then fixed in absolute ethanol, stained in crystal violet, and colonies with >50 cells counted. This process was repeated three times to generate the data presented here.

### Telomeric siRNA Knockdowns

As per our previous reports (62–64, 68), small interfering RNA (siRNA)-mediated knockdown of the telomere-binding proteins TRF1, TRF2, and POT1 was performed in the WTK1 lymphoblastoid cell line prior to shipment to BNL for irradiation. Cells were cotransfected (RNAiMAX; Life Technologies) with a pool of four individual siRNAs directed against each target protein. Specific siRNA sequences are as follows: for TRF1: 5′CAAAUUCUCAUAUGCCUUU3′, CAGUAGUAGUCCUUUGAUA, AGAGUAACCUAUAAGCAUG, and UACCAGAGUUAAAGCAUAU; TRF2: GAACAAGCGCAUGACAAUA, GCAAGGCAGCUACGGAAUC, GACAGUACAACCAAUAUAA, and CCGAACAGCUGUGAUGAUU; and POT1: GUAGAAGCCUUACGUGUUU, GAUAAAACAUCGUGGAUUC, GCAUAUCCGUGGUUGGAAU, and UAACUUGCCUGCUCUUUAG. Reduced protein levels were verified via Western blot 72 h post-transfection using monoclonal antibodies for TRF1 (Novus Biologicals 57-6), TRF2 (Novus Biologicals NB110-57130SS), and POT1 (Novus Biologicals NB500-176).

Depletion of hTERT and hTERC levels in MCF-7 and MCF-10A cells was achieved using prevalidated Silencer Select siRNAs purchased from Ambion (Life Technologies, hTERT: 4392420 and hTERC: 4390771). Non-target control (NTC) siRNA (Life Technologies, AM4611) was utilized as a negative control. The effectiveness of hTERT and hTERC depletion in reducing telomerase activity was verified using RT-qPCR TRAP.

### Cytogenetic Analyses

#### Chromosome-Orientation Fluorescence *In situ* Hybridization

Chromosome-orientation fluorescence *in situ* hybridization (CO-FISH) was employed to evaluate IR-induced chromosomal instability and performed as previously described (62, 70) with some modification. Following irradiation, cell cultures were incubated for various times, trypsinized, and subcultured into medium containing 5-bromo-2-deoxyuridine (BrdU, 10 μM; Sigma-Aldrich) for one cell cycle. Slides were stained with Hoechst 33258 (0.50 ng/μL; Sigma-Aldrich) for 15 min and exposed to 365 nm UV light (Stratalinker 2400) for 25 min. Following UV exposure, BrdU incorporated strands were digested with Exonuclease III (3 U/μL in provided reaction buffer; Promega) at room temperature for 10 min. Slides were hybridized with a Cy-3 conjugated (TTAGGG)_3_ PNA telomere probe (0.2 μg/mL; Applied Biosystems) at 37°C for 1.5 h, rinsed in 70% formamide at 32°C for 10 min, and dehydrated in another ethanol series before re-probing at 37°C for 2 h. Following the second hybridization, slides were rinsed with 70% formamide at 32°C for 15 min followed by 5 min rinse in PN buffer. Chromosomes were counterstained with DAPI (4,6-diamidine-2-phenylindole dihydrochloride; Vectashield, Vector Laboratories). Preparations were examined and images captured and analyzed using a Zeiss Axioskop2 Plus microscope equipped with a Photometrics Coolsnap ES2 camera and running Metavue 7.1 software.

#### Scoring Criteria

Telomere sister chromatid exchange was scored as a CO-FISH telomere signal split between the two chromatids of a metaphase chromosome, which were often of unequal intensity due to unequal SCE (71). Telomere fusion necessitates that telomeres of adjoining chromosomes/chromatids fuse into a single CO-FISH signal and the DAPI signal remain continuous (61). Telomere–DSB (T-DSB) fusion appears as single-sided (i.e., on only one chromatid of a mitotic chromosome) interstitial blocks of CO-FISH telomere signal (66, 67). Statistical analyses by Chi-square or Fisher’s exact test (Sigma Stat 3.5; Systat Software) was done to determine significance.

### Mutation Frequency Analysis

#### Mutation Assay

WTK1 lymphoblasts were treated with CHAT (10–5M 2′-deoxycytidine, 2 × 10^−4^M hypoxanthine, 2 × 10^−7^M aminopterin, and 1.75 × 10^−5^M thymidine; Sigma) for 2 days and CHT (CHAT without aminopterin) for 1 day to eliminate pre-existing TK^−^ mutants. Following CHAT treatment, cells were transfected with TRF1, TRF2, or POT1 siRNA and/or treated with the DNA-PKcs inhibitor Nu7026 (Sigma-Aldrich). Three days later, cells were irradiated with γ-rays or HZE particles. Two days after irradiation, when phenotypic expression of newly induced mutants was complete, the mutant fractions (MFs) were determined. For plating efficiency, 1 cell/well was seeded, or for scoring mutants, 2000 cells/well were seeded in the presence of 2 μg/mL trifluorothymidine (TFT; Sigma-Aldrich). Fresh TFT was added 11 days after plating, and plates were scored for positive or negative wells after 20 days. The MFs were calculated using the Poisson distribution, and statistical analyses were done by *t*-tests using Sigma Stat 3.5 (Systat Software).

### Telomere Length Analysis

#### Interphase Telomere Fluorescence *In situ* Hybridization

Samples were prepared using standard cytogenetic techniques as described previously with slight modifications (68, 72, 73). Briefly, cultured cell pellets were resuspended in 8 mL of 75 mM potassium chloride (KCl; hypotonic) and incubated for 30 min at 37°C. Following incubation, 1 mL of fixative (3:1 methanol acetic acid) was added, cells were pelleted at 1000 rpm for 5 min, resuspended in 6 mL fixative, and stored at −20°C. Fixed cell pellets were then washed and dropped onto glass slides for telomere fluorescence *in situ* hybridization (FISH), which was performed as described previously with modifications (74). Briefly, slides were treated with 100 μg/mL RNASE A in 150 mM NaCl, 15 mM sodium citrate buffer for 30 min at 37°C, dehydrated through an ethanol series (75, 85, and 100%), and denatured in a 70% formamide/2× saline sodium citrate (SSC) solution at 70°C for 2 min. A telomere peptide nucleic acid (PNA) probe (TTAGGG)_3_ labeled with Cy-3 was hybridized onto the slides at 37°C overnight. Slides were washed twice each in 50% formamide/2× SSC, 2× SSC, and 0.1% NP-40 in 2× SSC for 2.5 min each at 43°C. Finally, slides were mounted in Prolong Gold Antifade reagent (Invitrogen) containing 4′,6-diamidino-2-phenylindole, dihydrochloride (DAPI).

Image Z stacks were taken using a Zeiss Axio Imager.Z2 microscope, with a Coolsnap ES2 camera running Metamorph 7.7 (Molecular Devices). For each slide, 30–50 images were obtained, each consisting of 22, 0.2 μm stacks. Metamorph nearest neighbor deconvolution and stack compression functions were applied, followed by image thresholding (upper and lower threshold values were held consistent across experiment). Finally, a region of interest was created for each nucleus, and the intensities of individual telomeres obtained in metamorph. Fluorescence values in each batch of FISH were standardized to the fluorescence intensity of an LY-R mouse lymphoma cell pellet as an internal control. LY-R cells have long brightly staining telomeres and their use for standardization, which was adapted from Q-FISH (75), represents a means to accurately compare relative telomere lengths from run to run.

#### Senescence-Associated β-Galactosidase Assay

Irradiated and control cells were rinsed twice with PBS, fixed for 15 min in 4% paraformaldehyde at room temperature, rinsed with PBS and covered with freshly prepared β-galactosidase staining solution (Cell Signaling Cat #9806). Cells were incubated at 37°C in a dry incubator for 18 h, staining solution was aspirated and replaced with 70% glycerol, then imaged immediately on an EVOS digital microscope. Images were taken of each sample under phase contrast at 40×. Blinded subjective scoring of blue cells was used to quantify senescent cell fractions.

### Telomerase Analysis

#### Telomerase Activity

Telomerase activity was evaluated using the telomere repeat amplification protocol (TRAP) assay originally described by Herbert et al. (76) and adapted for quantitative real-time PCR by Hou et al. (77). Briefly, whole cell lysates were prepared from cultured cell pellets and lysed in cold MPER mammalian protein extraction buffer (Thermo Fisher) containing a protease inhibitor cocktail (Roche) and RNasin ribonuclease inhibitor (Promega) at a ratio of 100 μL of buffer per 1,000,000 cells. Lysates were cleared by centrifugation at 14,000 rpm for 10 min at 4°C and stored at −80°C. Protein concentration was determined using the Bradford Assay (Biorad).

The SYBR green master mix (Promega) included all components for the RTQ-PCR. Each well contained between 0.1 and 0.25 μg protein lysate, 50% volume SYBR green master mix, 0.2 μg T4 gene32 protein (New England Biolabs, Ipswitch, MA, USA), 0.1 μg of each primer TS (5′-AATCCGTCGAGCAGAGTT-3′) and ACX (5′-GCGCGG(CTTACC)^3^CTAACC-3′) (Integrated DNA Technologies), and RNase/DNase-free water to achieve a final well volume of 25 μL. The PCR and detection were performed on a CFX 96 (Biorad). In addition to the treatment samples, a series of controls were included on each plate: (1) no template control with TS primer only, (2) no template control with ACX primer only, (3) no template control with TS and ACX primers (used in normalization of samples), (4) heat inactivated control with template (protein lysate) and TS and ACX primers, and (5) HeLa cell lysate with TS and ACX primers (a positive control).

The RTQ-PCR program included the following steps: step 1, one cycle 25°C 20 min (telomerase elongates the TS primer by adding TTAGGG repeat sequences); step 2, one cycle 95°C 3 min (heat activation of the enzyme in the SYBR master mix); step 3, 40 cycles of 95°C 20 s, 50°C 30 s, and 72°C 1 min 30 s (PCR amplification allows for detection by real-time instrument); and step 4, 80 cycles 0.10 s per cycle (melt curve to ensure no primer dimer formation). Each sample was run in triplicate on a 96-well plate format allowing for an average Ct to be obtained per sample. Utilizing the average Ct value, the relative percent telomerase activity in each sample is calculated using the Delta Delta Ct method (2^−ΔΔCt^) (78). Briefly, to calculate the percent relative activity for each sample, first normalize the average sample Ct to the no template control with TS and ACX primers. This is referred to as the delta Ct value. The delta Ct value of each sample is subtracted from the delta Ct value of a chosen comparative sample, in this case a normal feline mucous membrane cell lysate, yielding a delta delta Ct value (ΔΔCt). Using the 2^−ΔΔCt^, a relative value is generated for each sample comparison and when multiplied by 100 is the relative percent of telomerase activity (RTA) of the sample compared to the control. The RTA can be compared between samples assayed across different plates. Results from two runs were averaged.

#### Telomerase Expression

Telomerase expression (hTERT and hTERC) was evaluated by quantitative reverse transcription PCR (qRT-PCR). Total RNA was harvested from irradiated and unirradiated samples using the Qiagen RNeasy kit (Qiagen). RNA was quantified using a Nanodrop 1000 spectrophotometer and reverse transcribed using the Verso cDNA kit (Thermo Scientific). Real-time PCR was performed using SYBR green master mix (Promega) according to the manufactures protocol and performed using a CFX 96 system (Biorad). The real-time cycle was as follows: cycle 1 at 95°C 15 min, cycle 2 (50×) step 1 at 95°C 15 s, step 2 at 58°C 30 s, and step 3 at 72°C 30 s. A melt curve was included to assess primer dimers and non-specific amplification as follows: cycle 3 at 95°C 30 s, cycle 4 at 55°C 30 s, and cycle 5 (80×) at 55°C 10 s. Primers were designed using the Primer3 program (79) using a published cDNA library for hTERT (80) and hTERC (80). hTERT primers were added (final concentration 300 nM) including a forward sequence: 5′CCATCAGAGCCAGCTTCACCT3′ and reverse sequence: 5′TCACCTGCAAATCCAGAAACA3′. hTERC primers were added (final concentration 300 nM) including a forward sequence: 5′AAGAGTTGGGCTCTGTCAGC3′ and reverse sequence: 5′TCCCACAGCTCAGGGAATC3′. Primers for transferrin receptor (TFRC) were included (final concentration 100 nM) as a housekeeping gene with the forward sequence: 5′CGCTGGTCAGTTCGTGATTA3′ and the reverse sequence: 5′GCATTCCCGAAATCTGTTGT3′. Relative hTERT and hTERC RNA expressions were analyzed using the 2^−ΔΔCt^ method.

#### Telomerase Activity Inhibition

Telomerase activity inhibition was accomplished using the small molecule inhibitor MST-312 (Sigma), also known as telomerase inhibitor 1× (81). Briefly, MST-312 was solubilized at concentrations recommended by the manufacturer in sterile DMSO and stored at −20°C for no more than 1 month prior to use. Dose response for inhibition of telomerase activity was established, and MST-312 (1–3 μM) was added to cultures 6 h prior to experimentation.

#### Thiazolyl Blue Tetrazolium Bromide (MTT) Assay

Thiazolyl blue tetrazolium bromide (MTT) assay was used to evaluate potential cytotoxic effects of the MST-312 telomerase inhibitor as described previously (82). Briefly, 2000 cells/well of a 96-well plate were seeded 24 h prior to addition of inhibitor. Media were removed and replaced with fresh media containing varying concentrations of MST-312 or an equivalent DMSO control. Cells were incubated in the presence of inhibitor for 48 or 72 h. At the time of analysis, media was once again removed from wells, and cells were resuspended in fresh media containing 0.5 mg/mL MTT reagent and incubated at 37°C for 3.5 h. Following incubation, media was removed and 150 μL MTT solvent (4 mM HCl, 0.1% NP-40, all in isopropanol) was added to each well and set to agitate on a shaker at room temperature for 15 min. After agitation, plates were read on a Modulus Microplate reader (Turner Biosystems) at 600 nm absorbance.

### Stem Cell Analyses

#### Immunophenotyping

Putative human mammary epithelial CSCs were identified based on the expression of CD44^+^/CD24^low/−^ surface markers (83–85). Putative CSCs from human hematopoietic cell lines were identified based on the expression of CD34^+^/CD38^low/−^ or CD34^+^ expression alone (86). All analyses were performed on a CyAn ADP Analyzer with nine-color capability (Beckman Coulter CY20130) located at the Colorado State University Veterinary Teaching Hospital. Monolayers MCF-7 and MCF-10A mammary epithelial cells or hematopoietic cells in suspension were dissociated and stained for marker expression. Briefly, ~300,000 cells were dissociated from cell culture surface using 0.25% trypsin-EDTA, pelleted, washed, and resuspended in 30 μL flow cytometry wash buffer (1× PBS, 1% FBS, and 1% penicillin/streptomyocin), 6 μL of FITC-conjugated mouse monoclonal antihuman CD44 antibody (BD Pharmingen #555478), and 6 μL of Pe-conjugated mouse monoclonal antihuman CD24 antibody (BD Pharmingen #555428). Cells were incubated for 30–60 min in the dark at 4°C, then pelleted and resuspended in 500 μL cold 1× PBS and kept on ice until analysis. Analysis gates were established using cells from unstained controls and antimouse Ig,κ antibody capture beads (BD Pharmingen #552843). For lymphoblastoid suspension cultures, cells were stained as above with 6 μL of direct PE-conjugated mouse monoclonal antihuman CD34 antibody (BD Pharmingen #555822) and 6 μL of direct FITC-conjugated mouse monoclonal antihuman CD38 antibody (BD Pharmingen #560982).

#### Aldefluor Assay

Enhanced ALDH activity, an accepted marker of SCs, was detected using the ALDEFLUOR Kit (Stem Cell Technologies) as described previously (29). Briefly, ~300,000 MCF-7 or MCF-10A cells were trypsinized, resuspended in aldefluor buffer, and incubated with aldefluor reagent (both provided and as recommended by manufacturer). Samples from all treatment groups were also treated with the ALDH inhibitor DEAB (Stem Cell Technologies) and utilized as negative controls to establish gating. Cells were pelleted, aspirated, resuspended in buffer containing efflux inhibitor, and analyzed on a flow cytometer.

#### Mammosphere Assay

Evidence of SC character was also evaluated in MCF-7 and MCF-10A mammosphere cultures and their respective sorted populations, which were plated in low bind cell culture plates (Nunc) using Mammocult Media (Stem Cell Technologies). Cells were plated into 96-well plates at limiting dilutions, then allowed to form spheres for up to 10 days with fresh media supplementation every 3 days. Sphere formation was evaluated on day 10 using an inverted bright field microscope, and spheres with a size >100 μM in diameter were scored.

## Results

### Depletion of Telomeric End-Capping Proteins Increases Radiation-Induced Mutation Frequencies and Chromosomal Instability

**Figure 1:**
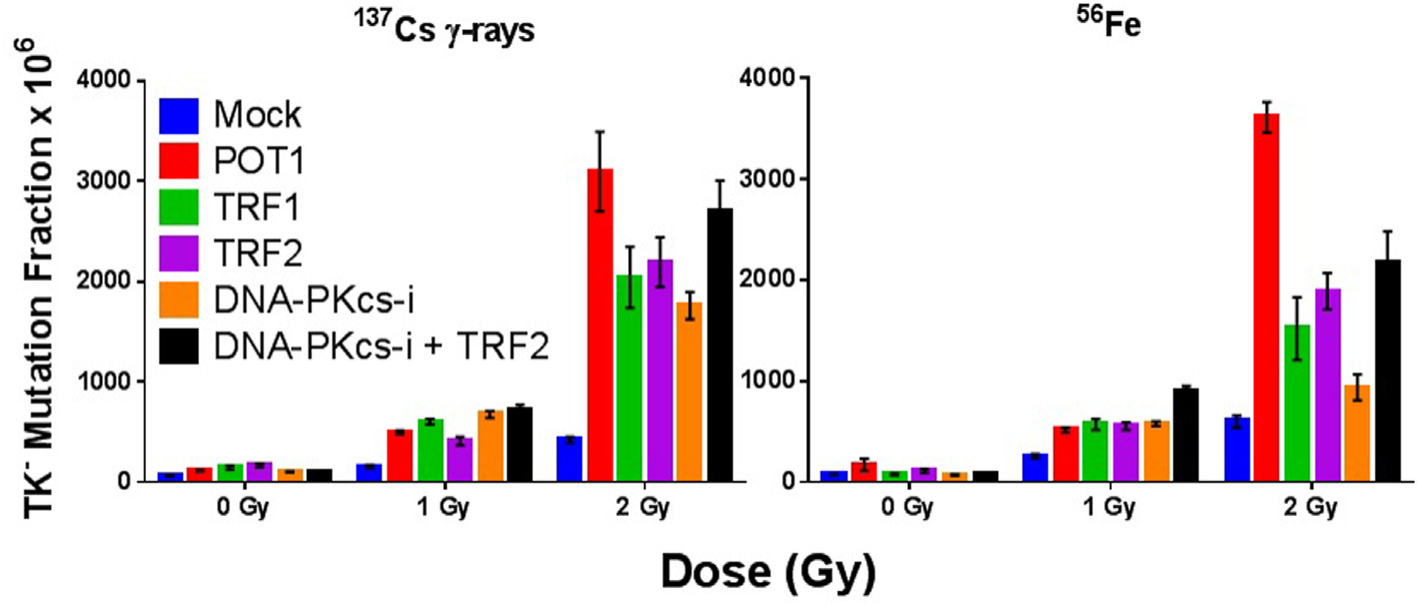
**Compromise of telomeric end-capping function elevates spontaneous and radiation-induced mutagenesis**. TK^−^ mutation frequency (MF) in WTK1 lymphoblastoid cells exposed to 0, 1, or 2 Gy γ-rays or ^56^Fe ions at 1 GeV/n following siRNA knockdown of telomere-binding proteins POT1, TRF1, and TRF2, and/or inhibition of DNA-PKcs kinase activity.

**Figure 2:**
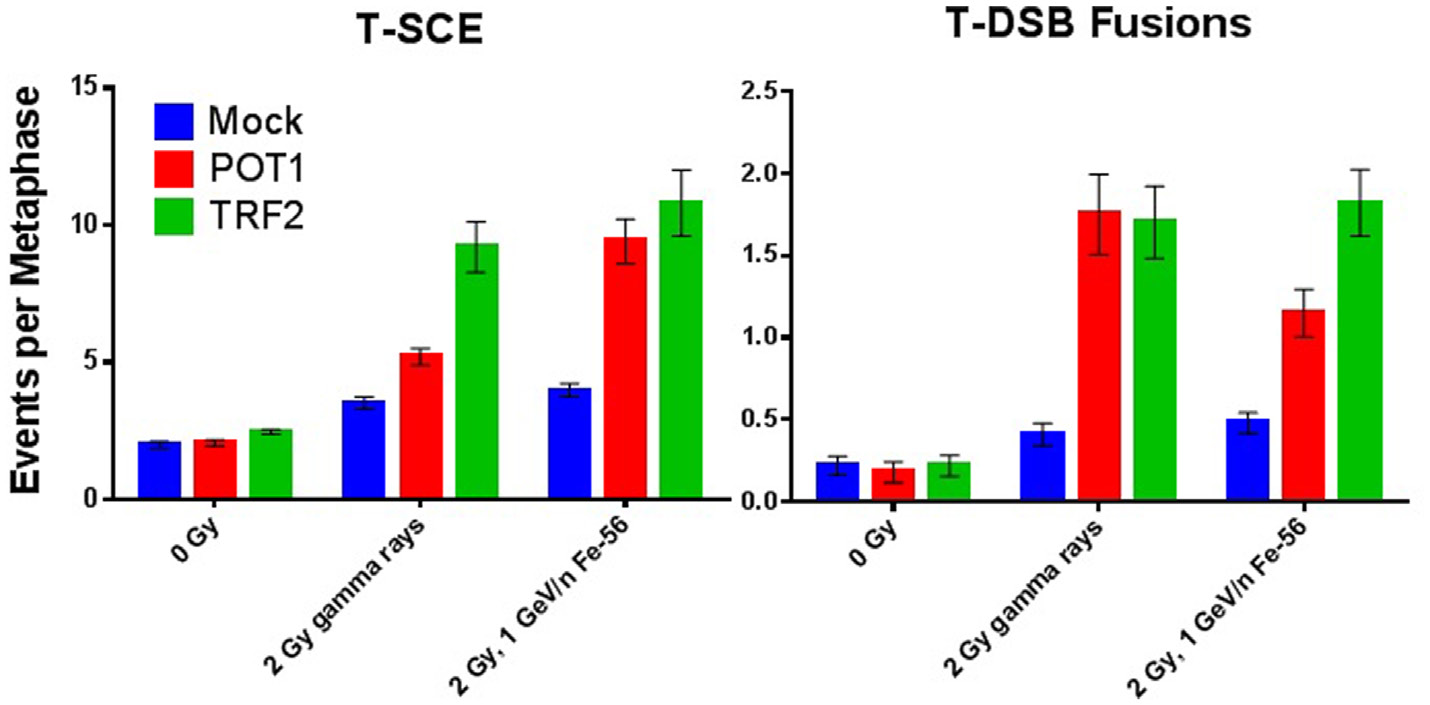
**Compromise of telomeric end-capping function increases radiation-induced T-SCE and telomere–DSB fusion events**. T-SCE and telomere–DSB fusion frequencies in WTK1 lymphoblastoid cells exposed to 0 Gy, 2 Gy γ-rays, or 2 Gy 1 GeV/n ^56^Fe ions following siRNA knockdown of telomere-binding proteins POT1 or TRF2.

We have shown that TRF2 fails to colocalize with IR-induced DSBs and so is not an “early responder” to such DNA damage (87). However, siRNA depletion of any of the directly binding end-capping telomere proteins TRF1, TRF2, or POT1 resulted in elevated spontaneous and IR-induced mutation frequencies (MF) at the heterozygous TK locus in human lymphoblastoid cells (WTK1) following both γ-ray and 1 GeV/n ^56^Fe ion exposures (Figure 1). Overall, IR-induced MFs upon telomere protein depletion were similar both quantitatively and qualitatively, although POT1 depletion resulted in the highest elevation of MF (significant at 2 Gy Fe). No statistically significant differences in MF between γ-rays and 1 GeV/n ^56^Fe ions (HZE) were observed in these experiments. However, in this same lymphoblast mutation system, an RBE for Fe of ~3 was recently suggested; this resulted from utilizing a different, more immediate plating protocol that facilitated recovery of more mutants following HZE exposure (88). Therefore, we can now surmise that the MFs for Fe in the earlier experiments reported here are likely two to threefold higher than shown. This in turn would imply important differences for HZE exposures in the context of telomere deficiencies. Interestingly, telomere deficiencies consistently resulted in higher MF than inhibition of DNA-PKcs kinase activity, a well-characterized contributor to DNA repair, providing additional support for the significance of telomere proteins in the DDR. Furthermore, MF in the context of combined TRF2 knockdown and inhibition of DNA-PKcs kinase activity was not additive, suggesting that they act in the same pathway. These results are consistent with the proposition that TRF2 prevents C-NHEJ-mediated end fusion, while DNA-PK thwarts alternative-NHEJ at telomeres; thus, telomeres are protected by a “lock with two bolts” (89). Curvilinear dose responses for individual knockdowns were also suggested (Figure 1), indicating intertrack interaction of multiple lesions at higher doses, and likely reflecting additional interactions between dysfunctional telomeres and IR-induced DSBs (T-DSB fusions) (66); such a supposition is supported by increased frequencies of these events at 2 Gy (Figure 2).

The contribution of telomere end-capping function to IR-induced chromosomal instability was also evaluated, as per our previous works (61–63, 67). T-SCE is a recognized marker of unregulated telomeric recombination events (71), whereas telomere–DSB (T-DSB) fusion events result from inappropriate end joining (66). Quantification of T-SCE and T-DSB fusion frequencies associated with siRNA knockdown of TRF2 or POT1 in WTK1 cells, both spontaneously and following acute exposure to either 2 Gy γ-rays or 2 Gy 1 GeV/n ^56^Fe ions is shown (Figure 2). Successful siRNA knockdown of TRF1, TRF2, and POT1 in WTK1 cells 72 h post-transfection was verified by Western blot (Figure S1 in Supplementary Material).

Depletion of either TRF2 or POT1 elevated T-SCE frequencies following both γ-ray and HZE 2 Gy exposure as compared to 0 Gy controls. No statistically significant difference between γ-rays and HZE was observed with TRF2 deficiency. However, HZE was much more effective than γ-rays at inducing T-SCE in the context of POT1 deficiency, consistent with the demonstrated role of POT1 during replication (90, 91). Depletion of either TRF2 or POT1 also elevated T-DSB fusion events following 2 Gy γ-ray or HZE exposure (as compared to 0 Gy controls). Again, no statistically significant difference between γ-rays and HZE was observed with TRF2 deficiency, a finding consistent with TRF2’s role in suppressing ATM at telomeres (90). Interestingly, with POT1 deficiency, HZE was less effective at inducing T-DSB than γ-rays, supportive of these events not being replication dependent, but rather NHEJ mediated, as previously shown (67). Together, these results convincingly demonstrate that telomeric proteins influence the DDR/repair following IR exposure.

### Ionizing Radiation Exposure Increases Telomerase Activity

**Figure 3:**
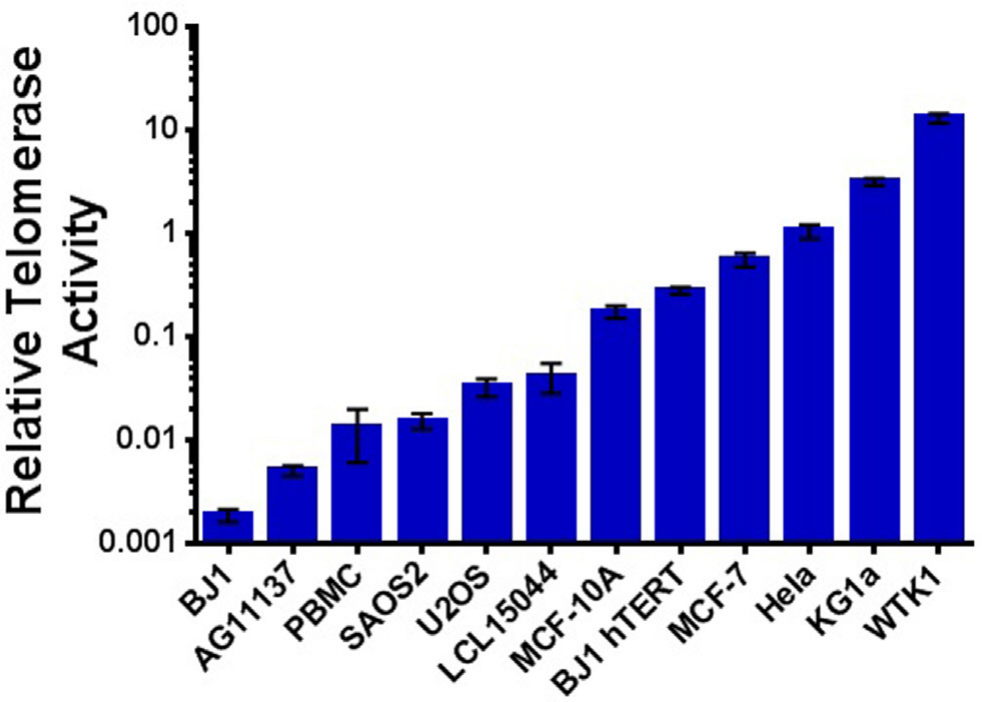
**Telomerase activity in cancer and non-cancer cell lines**. Relative telomerase activity as determined using the qRT-PCR-based TRAP assay and arranged in order from least to greatest background level; normal human PBMCs are also included. Consistent with expectation, a natural break occurs between those with very low/low activity (LCL15044 and to the left; normal, ALT, and often considered negative) and those with higher levels of telomerase (MCF-10A and to the right; immortalized, transformed, and cancer). Data are represented on a log scale, and all values are reported as telomerase activity relative to HeLa cells.

Previous reports have demonstrated elevated telomerase activity following IR exposure; however, results are often conflicting in regard to dose, dose rate, radiation quality, method of telomerase activity measurement, and cell line examined. Therefore, we sought to more clearly characterize telomerase activity in response to a variety of IR exposures in both tumor and non-tumor cells. We selected panels of human mammary epithelial and hematopoietic cell lines representing a wide range of inherent/background levels of telomerase activity (high/low/very low) that included cancer (MCF-7 and KG1a), non-tumorigenic immortalized [spontaneously (MCF-10A) or via EBV (WTK1)], and normal primary mammary (AG11137) and low passage lymphoblastoid (LCL15044) cell lines. Telomerase activity was evaluated relative to the telomerase-positive HeLa cell line (Figure 3). The ALT cell lines U2OS and SAOS2 (telomerase-independent maintenance of telomeres) and BJ-1 primary foreskin fibroblasts (very low telomerase activity) were utilized as negative controls, and hTERT immortalized BJ-1 fibroblasts (BJ-1-hTERT) were also used as an internal control.

**Figure 4:**
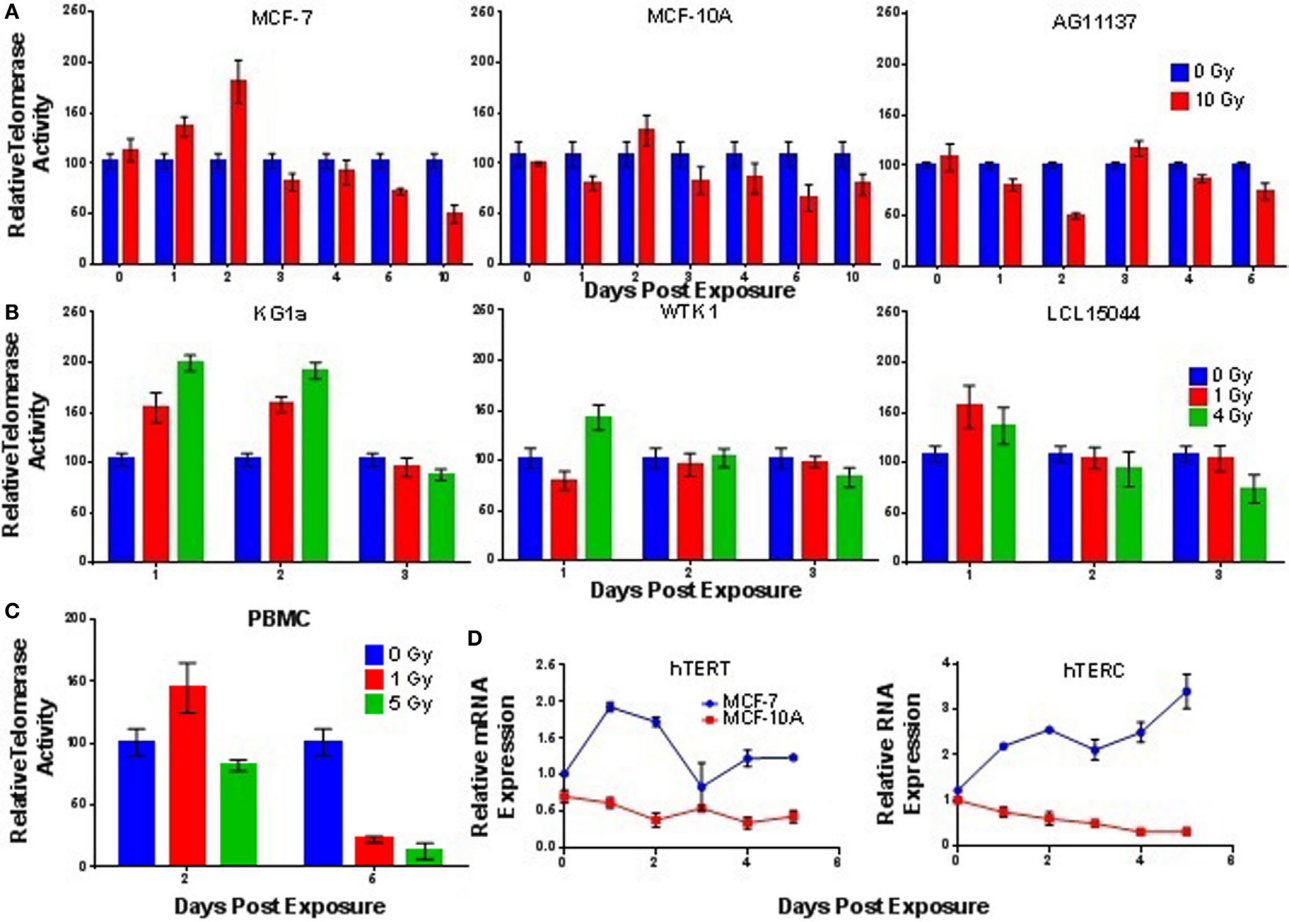
**Telomerase activity is elevated at early times following acute γ-ray exposure**. Telomerase activity was assessed as a function of time in panels of **(A)** cultured mammary epithelial cell lines, **(B)** hematopoietic cell lines, and **(C)** stimulated peripheral blood mononuclear cells (PBMCs) following acute γ-ray exposures: 10 Gy (mammary epithelial), 1 and 4 Gy (hematopoietic), and 1 and 5 Gy (PBMCs). In general, cell lines with higher levels of background activity experienced higher, more significant elevation of activity 24–48 h postexposure, the notable exception being normal PBMCs (very low telomerase activity; significant IR-induced elevation). Telomerase activity in irradiated cells is reported relative to unirradiated control samples collected at the same time point. **(D)** Time course of hTERT mRNA and hTERC levels in MCF-7 and MCF-10A cells following acute 10 Gy exposure.

Acute exposure to γ-rays (10 Gy) prompted significantly elevated levels of telomerase activity within 24–48 h in the mammary MCF-7 cells (high inherent telomerase activity, 24 h; *p* = 0.0140, 48 h; *p* = 0.0011); a slight, but non-significant (*p* = 0.2473) elevation of telomerase activity was also observed in MCF-10A cells (lower inherent telomerase activity) at 48 h (Figure 4A). In contrast, a significant reduction of telomerase activity (*p* = 0.0001) was observed 48 h postexposure in the AG11137 primary mammary epithelial cells (very low inherent telomerase activity). Evaluation of telomerase RNA expression in MCF-7 and MCF-10A following acute 10 Gy γ-ray exposure, specifically mRNA levels of the catalytic subunit hTERT and expression levels of the RNA template hTERC, revealed that hTERT expression in MCF-7 cells was significantly increased in the same timeframe that telomerase activity was elevated (24–48 h, 24 h; *p* = 0.0001, 48 h; *p* = 0.0001); hTERC levels in MCF-7 remained significantly elevated for up to 5 days (24 h; *p* = 0.0001, 120 h; *p* = 0.0011). In MCF-10A, hTERT expression was significantly decreased 72–120 h postexposure (72 h; *p* = 0.0326, 120 h; *p* = 0.0337), and hTERC expression in MCF-10A was significantly decreased 72–120 h (72 h; *p* = 0.0005, 120 h; *p* = 0.0001; Figure 4D).

The highly telomerase-positive KG1a cell line displayed significantly increased levels of telomerase activity 24–48 h postexposure (1 Gy, 24 h; *p* = 0.0024, 48 h; *p* = 0.0001, 4 Gy, 24 h; *p* = 0.0019, 48 h; *p* = 0.0001; Figure 4B), which was dose dependent (1 and 4 Gy γ-rays). A significantly elevated level of telomerase activity was also observed in the WTK1 cell line (high inherent telomerase activity) at 24 h postexposure (*p* = 0.0143), but only at 4 Gy. The low passage transformed normal lymphoblastoid cell line LCL15044 (very low telomerase activity) showed elevated telomerase activity at 24 h post 1 and 4 Gy exposure, but neither rose to the level of significance. Interestingly, normal PBMCs (very low telomerase activity) showed elevated activity 48 h post 1 Gy acute γ-ray exposure, which was significant (*p* = 0.0394; Figure 4C).

**Figure 5:**
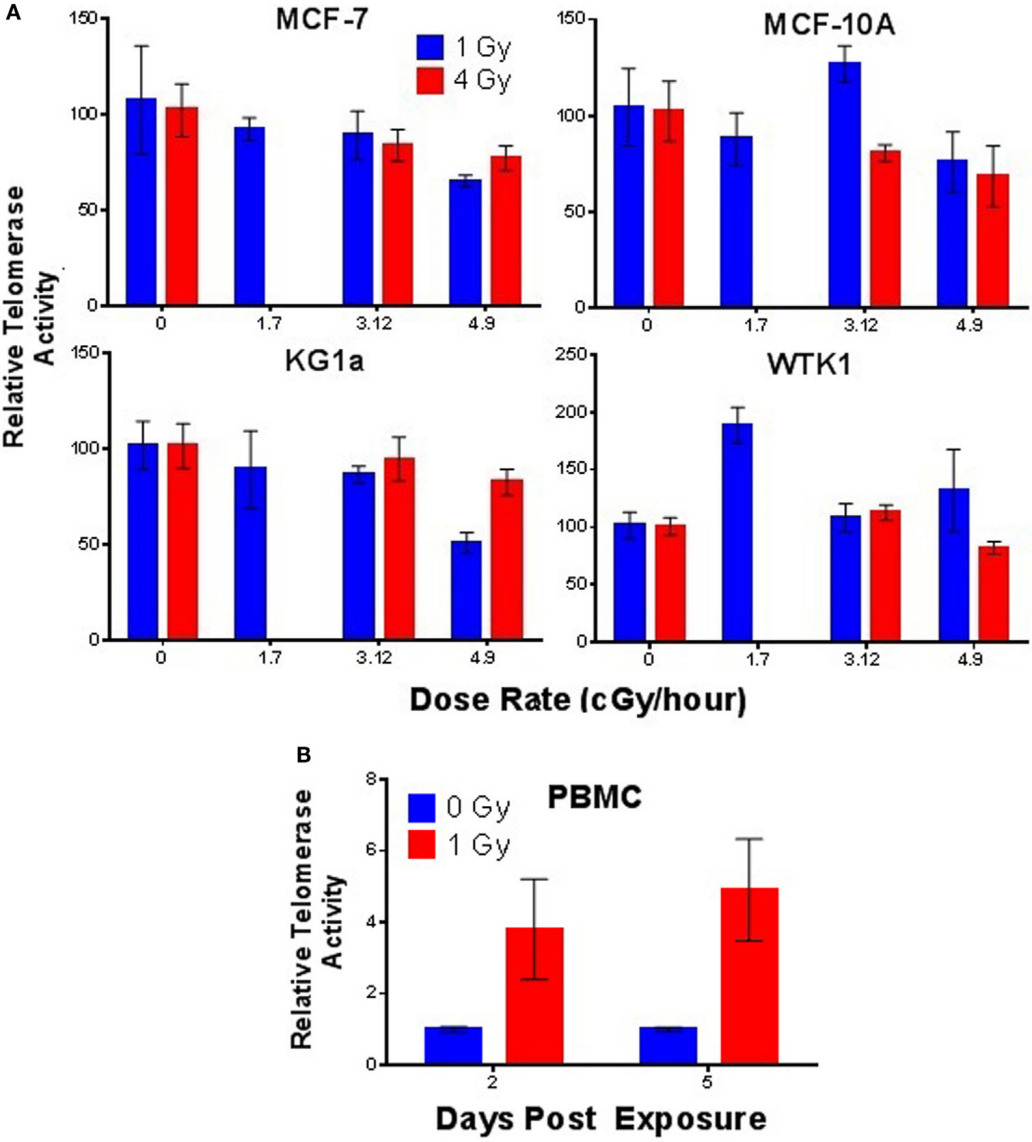
**Telomerase activity following low dose rate γ-ray exposures**. **(A)** Telomerase activity was assessed in MCF-7, MCF-10A, KG1a, and WTK1 (relatively high levels of telomerase) chronically exposed to γ-rays at cumulative doses of 1 or 4 Gy at dose rates of 1.7, 3.12, or 4.9 cGy/h. In general, no elevation of telomerase activity was observed, with the exception of WTK1 at 1 Gy, 1.7 cGy/h. **(B)** In contrast, telomerase activity in stimulated PBMCs (very low telomerase activity) exposed to a total dose of 1 Gy delivered at low dose rate of 4.9 cGy/h, was significantly elevated (days 2 and 5). Data are expressed as telomerase activity relative to unirradiated controls within each group.

Next, we sought to determine if telomerase activity was elevated in response to chronic low dose rate (LDR) γ-ray exposure. MCF-7, MCF-10A, KG1a, and WTK1 cells (relatively high telomerase activity) were incubated under chronic LDR conditions to total doses of 1 or 4 Gy, delivered at dose rates of 0, 1.17, 3.12, and 4.98 cGy/h. Telomerase activity was not significantly elevated at either 1 or 4 Gy total dose, at any dose rate, in any cell line examined (Figure 5A). These results imply that in contrast to acute exposures, elevation of telomerase activity is not triggered by low LET, LDR exposures. However, normal human PBMCs exposed to chronic LDR radiation did respond with elevated levels of telomerase activity relative to unirradiated controls (Figure 5B).

### Telomere Length Is Shortened Despite Elevated Telomerase Activity Postexposure

**Figure 6:**
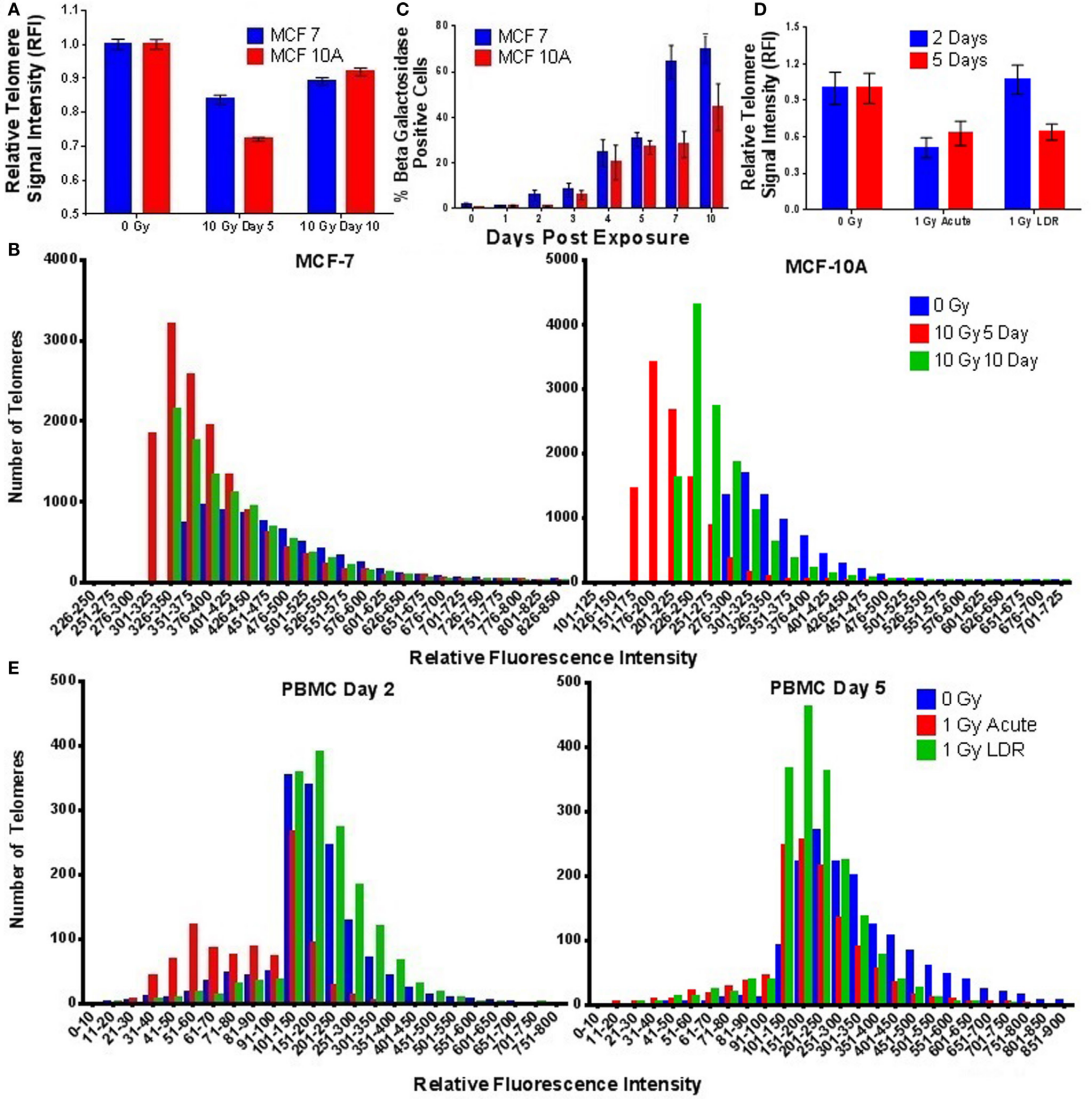
**Ionizing radiation-induced telomere shortening in mammary epithelial cells and stimulated PBMCs**. **(A)** Telomere length was significantly shortened in MCF-7 and MCF-10A cells at the population level 5 days postacute γ-ray exposure (10 Gy). By 10 days, telomere length had increased but still remained significantly shortened compared to unirradiated controls. **(B)** Histograms of individual telomere lengths in MCF-7 and MCF-10A cells reveal significant shortening of all telomeres at days 5 and 10 postexposure. **(C)** Time course of senescence associated β-galactosidase staining in MCF-7 and MCF-10A cells following acute γ-ray exposure (10 Gy). **(D)** Telomere length was significantly shortened in stimulated PBMCs at 2 and 5 days postacute γ-ray exposure (1 Gy) and at 5 days post-LDR γ-ray exposure (1 Gy) delivered at a dose rate of 4.9 cGy/h (24 h). **(E)** Histograms of individual PBMC telomere lengths following acute and LDR exposures (1 Gy) at 2 and 5 days.

To evaluate the effect of elevated telomerase activity post-IR exposure, we assessed telomere length in dividing MCF-7 and MCF-10A cell populations at 5 and 10 days after a single acute dose (10 Gy γ-rays). Unexpectedly, telomere length was significantly shortened at the population level 5 days postexposure in both MCF-7 (*p* = 0.0001) and MCF-10A (*p* = 0.0003) (as compared to 0 Gy controls), despite the observed elevation of telomerase activity 24–48 h after exposure (Figure 6A). There was no change in telomere length immediately following a 10 Gy dose of γ-rays, ruling out the possibility of IR-induced changes in telomere/probe-binding affinity (not shown). Furthermore, histogram analysis of individual telomere lengths demonstrated that IR exposure shortened all of the telomeres in the population (i.e., shifted the entire distribution; Figure 6B), suggesting that IR does not “target” the shortest telomeres, those presumed to be more radiation sensitive. The observed post-IR telomere shortening also corresponded with a significant increase in senescent cells (SA-Beta gal positive) at day 5, which remained significantly elevated until at least day 10 (Figure 6C). This finding is consistent with well-documented IR-induced senescence; our results suggest an underlying contribution of telomere shortening and/or the inability of telomeres to repair themselves postexposure (92–94). Furthermore, by day 10 postexposure, telomere length in the surviving cell population began to recover, despite reduced levels of telomerase activity during this same time period. These results support the notion that following IR exposure, telomerase is acting outside of its canonical role in elongating telomeres. Telomere length was also decreased in PBMCs following an acute exposure (1 Gy) of γ-rays at both 2 and 5 days (2 days; *p* = 0.0328, 5 days; *p* = 0.0362; Figure 6D). Interestingly, a similar decrease in telomere length was observed following a 1 Gy LDR exposure at 5 (*p* = 0.0250) (but not 2) days (Figure 6E).

### Elevation of Telomerase Activity Precedes IR-Induced Enrichment of Putative Stem Cell Populations

**Figure 7:**
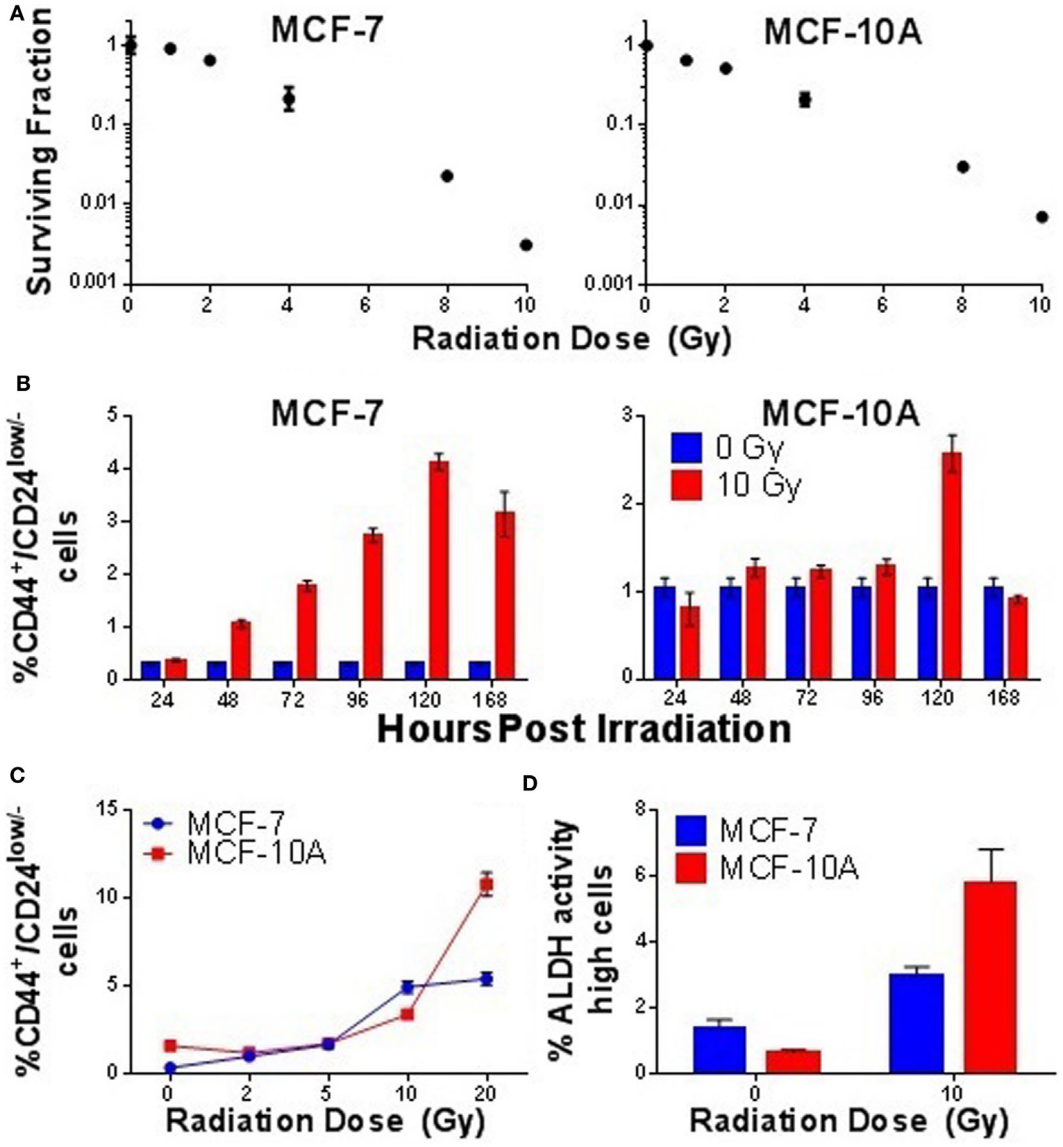
**Ionizing radiation-induced enrichment of putative mammary stem cell populations**. Responses of MCF-7 and MCF-10A mammary epithelial cells to acute γ-ray exposure (10 Gy). **(A)** Clonogenic survival curves and IR dose response. **(B)** Time course evaluation of putative mammary stem cell compartments. **(C)** Radiation dose response of putative mammary stem cells 5 days postexposure. **(D)** Quantification of ALDH high cells 5 days postexposure, confirming stem cell nature of sorted CD44^+^/CD24^−^ populations.

As normal SC and CSC populations generally possess higher telomerase activity than their non-stem counterparts, and IR-induced enrichment of putative SC populations in mammary carcinoma cells has been reported by multiple groups (23, 28, 29, 31, 83), we hypothesized that observations of elevated telomerase activity following IR exposure may result from the enrichment of CSC populations. Therefore, we examined enrichment of putative CD44^+^/CD24^low/−^ SC populations with time in MCF-7 and MCF-10A following an acute 10 Gy γ-ray exposure. At the therapeutically relevant dose of 10 Gy, survival in both cell lines was determined to be <1%, even when using a delayed plating method (Figure 7A). However, despite this low number of survivors, a significant enrichment in the percentage of CD44^+^/CD24^low/−^ MCF-7 cells began to emerge approximately day 2 post exposure (*p* = 0.0001), peaked at day 5 (*p* = 0.0001), and remained elevated at day 7 (*p* = 0.0001; Figure 7B). Interestingly, IR-induced enrichment of CD44^+^/CD24^low/−^ cells in MCF-10A cells also occurred at day 5 postexposure (*p* = 0.0001); however, unlike MCF-7 cells, enrichment of putative SCs in MCF-10A was very abrupt. Furthermore, IR-induced enrichment of mammary CD44^+^/CD24^low/−^ cells at day 5 in MCF-7 displayed a dose response and appeared to have a threshold dose of >5 Gy in MCF-10A, meaning that it did not significantly occur at doses lower than 5 Gy (Figure 7C). Stem-like character of radiation-enriched SC populations was further verified using the aldefluor assay, in which significant enrichment of ALDH^high^ cells was observed 5 days post acute 10 Gy γ-ray exposure (MCF-7; *p* = 0.004, MCF-10A; *p* = 0.001; Figure 7D); mammospheres were also generated from sorted populations, providing additional confirmation of stemness in the CD44^+^/CD24^low/−^ populations (not shown). Counter to our initial hypothesis, IR-induced elevation of telomerase activity preceded the observed enrichment of SC compartments, indicating that they are not directly correlated (i.e., are not one in the same), once again suggesting that telomerase is acting in non-canonical ways, likely having to do with SCs, and in agreement with previous reports (19, 38). Representative scatter plots of MCF-7 and MCF-10A cells immunotyped using CD44^+^/CD24^low/−^ antibodies or the aldefluor assay are shown (Figure S2 in Supplementary Material).

**Figure 8:**
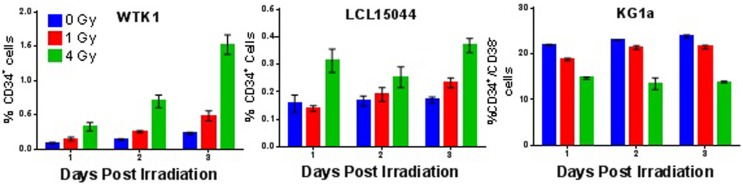
**Ionizing radiation-induced enrichment of putative hematopoietic stem cell populations**. Time course analysis depicting percentages of CD34^+^ (WTK1 and LCL15044) and CD34^+^/CD38^−^ (KG1a), 1, 2, and 3 days postacute γ-ray exposure (0, 1, or 4 Gy).

To determine whether the IR-induced enrichment of putative SC populations observed in mammary epithelial cells is a more general phenomenon that might also occur at lower doses, we examined expression of the CD34^+^/CD38^−^ immunotype in the hematopoietic cell line KG1a and the CD34^+^ immunotype in WTK1 and LCL15044 cell lines 1–3 days post 1 or 4 Gy γ-ray acute exposure (Figure 8). Interestingly, significant increases in CD34^+^ populations were observed in both WTK1 and LCL15044 lymphoblastoid cell lines (>99.8% CD34^−^ in unirradiated conditions) following either a 1 or 4 Gy exposure for up to 3 days. In contrast, KG1a cells, which possessed a much higher background compartment of CD34^+^/CD38^−^ cells (20–30%), demonstrated a dose-dependent decrease in CD34^+^/CD38^−^ levels with IR exposure (Figure 8). This result indicates that radiation does not induce enrichment of putative SC populations in all cell lines, cancer types, or tissues, which may, at least to some degree, be dependent on background levels.

### Telomerase Activity Is Required for IR-Induced Enrichment of Putative CSC Populations

**Figure 9:**
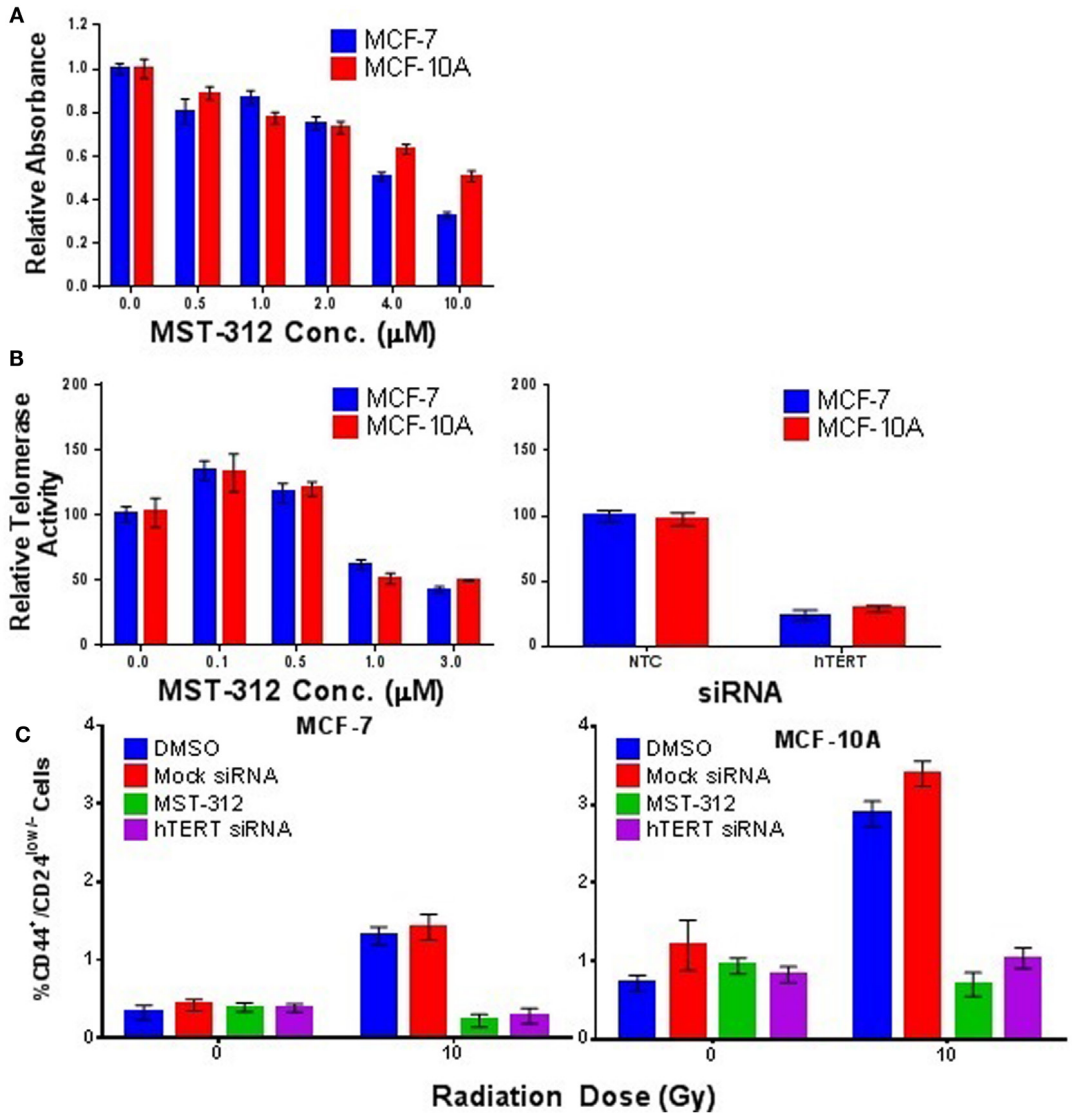
**Telomerase activity is required for IR-induced enrichment of putative, CD44^+^/CD24^low/−^ putative mammary stem cell populations**. **(A)** MTT assay establishing relative toxicity of the telomerase inhibitor MST-312 in MCF-7 and MCF-10A cells. **(B)** MST-312 reduced telomerase activity in a dose-dependent fashion in both cell lines. Treatment with siRNA targeted to the catalytic subunit hTERT also significantly reduced telomerase activity; siRNA targeted to hTERC did not (not shown). **(C)** Reduction of telomerase activity via either telomerase inhibition (MST-312) or siRNA knockdown (hTERT) prevented IR-induced enrichment of CD44^+^/CD24^low/−^ cell populations 5 days postacute γ-ray exposure (10 Gy).

To further investigate the role of telomerase in promoting IR-induced enrichment of putative SC populations, we employed both a small molecule inhibitor of telomerase activity (MST-312) and siRNA depletion of the catalytic subunit (hTERT) of telomerase. MCF-7 and MCF-10A cells were treated with MST-312 at non-cytotoxic concentrations as determined by MTT assay (Figure 9A), which resulted in significant and dose-dependent decreases in telomerase activity (Figure 9B). Subsequent exposure to an acute dose of 10 Gy γ-rays and incubation for 5 days demonstrated that inhibition of telomerase activity effectively blocked IR-induced putative SC enrichment in both MCF-7 and MCF-10A (Figure 9C). This finding was further substantiated utilizing siRNA directed against hTERT, which also significantly reduced the level of telomerase activity in both MCF-7 and MCF-10A (Figure 9B). Consistent with results using the inhibitor (MST-312), reduction of telomerase activity via depletion of hTERT also blocked IR-induced putative SC enrichment in both MCF-7 and MCF-10A (Figure 9C). As expected, siRNA depletion of the RNA subunit, hTERC, did not reduce telomerase activity, nor did it block SC enrichment (not shown). Together, these data provide strong evidence in support of the necessity of telomerase activity for IR-induced enrichment of CD44^+^/CD24^low/−^ putative mammary CSC populations postexposure.

## Discussion

The results reported here provide valuable insight into the critical roles telomeres and telomerase play in the radiation response and thereby support further exploration for their roles in the context of both radiotherapy and IR-induced carcinogenesis. Specifically, we have assessed the roles telomeres play in maintaining genomic stability following IR exposure, elaborated on the role telomerase plays in cell survival and repopulation postexposure, and identified a potentially targetable role of telomerase activity in cells exposed to therapeutically relevant doses of low LET radiation.

Disruption of telomeric end-capping via siRNA depletion of end-binding proteins TRF1, TRF2, or POT1 increased IR-induced mutation frequencies and chromosomal instability. Of relevance in this regard are reports of decreased expression of TRF2 associated with increased breast cancer malignancy (95), as well as the demonstration of telomere fusions in early human breast carcinoma (96). Furthermore, our finding of telomere uncapping with POT1 deficiency is consistent with POT1 mutations identified in a subset of patients with chronic lymphocytic leukemia (CLL), which were associated with increased levels of chromosomal fusions involving telomeres (97). A particularly relevant recent report specifically associated various POT1 variants with telomere length and radiosensitivity in colon and gastric adenocarcinoma (98). Our results provide additional support for the view that in addition to critical telomere shortening, telomeres rendered dysfunctional by virtue of deficiencies in telomeric proteins and the end-capping failure that ensues, also contribute to the carcinogenic potential of radiation exposure.

It is also noteworthy that while deficiencies in TRF1 or TRF2 appeared similar to those associated with NHEJ deficiency in regard to telomere instability, which presumably occurred via ATM-mediated classic NHEJ (99), the response of cells to IR exposure in the setting of POT1 knockdown differed. Not only did POT1 depletion result in significantly higher mutation frequencies in response to γ-rays and ^56^Fe ions (2 Gy; relative to TFR1 and TRF2 knockdown) but also a very different pattern of chromosomal instability was observed. Specifically, while TRF1 and TRF2 knockdown resulted in elevated frequencies of both T-SCE and T-DSB fusion events in response to both γ-rays and ^56^Fe ions, POT1 knockdown displayed a higher level of T-SCE (HR mediated) events in response to ^56^Fe ions relative to γ-rays, with the opposite being true for T-DSB (NHEJ mediated) events. These observations are consistent with the proposed roles for POT1 in suppressing ATR at telomeres (90) and facilitating a RPA-to-POT1 switch (91) during replication, and thereby suppressing telomeric recombination, here particularly in response to the complex damage induced by high LET radiation exposure. Taken together with the reported association of POT1 variants with radiosensitivity and colon and gastric adenocarcinoma (98), our results suggest that heavy ion radiation therapy may be particularly effective in treating these cancers.

Consistent with previous reports, we also demonstrate that telomerase activity is an IR-inducible function. We elaborate that *in vitro*, this phenomenon appears to be peculiar to cell lines with high background levels of telomerase activity (e.g., cancer and potentially SCs), and further that increased telomerase activity appears to be primarily an acute dose response, as LDR γ-ray exposures (at least at 1 and 4 Gy cumulative doses) did not elevate telomerase activity in the cell lines examined. Importantly, however, telomerase activity was elevated in PBMCs post-LDR exposure. Expression analysis of the hTERT mRNA and the hTERC RNA component of telomerase in MCF-7 cells coincided (temporally) with the elevations of telomerase activity, suggesting that this process may be transcriptionally regulated. Expression of telomerase subunits in MCF-10A cells was dramatically different in that both hTERT mRNA and hTERC steadily decreased from 1 to 5 days postexposure, a finding consistent with the absence of significant elevation of telomerase activity in this same timeframe. It is also important to appreciate, however, that although not significant, increases in telomerase activity were observed in the non-tumor MCF-10A and normal LCL15044 mammary epithelial cell lines following acute IR exposure, indicating that telomerase may indeed be induced, but the low background level of activity in these cell lines may yield such an increase relatively insignificant. Additionally, evaluation of telomerase activity in stimulated normal human PBMCs (low background level) revealed a significant increase following an acute dose (1 Gy), 2 days postexposure. Also in contrast to cultured cells, a significant increase in telomerase activity was observed in stimulated PBMCs 2 and 5 days post-LDR exposure (total 1 Gy delivered at a dose rate of 4.9 cGy/h). One potential explanation for this could be the more heterogeneous nature of cells in stimulated peripheral blood (including stem/progenitor cells) creating a disparate signaling environment relative to the more homogenous cells found in cultured lines. These findings suggest that IR-induced changes in telomerase activity are relevant to LDR environmental exposures *in vivo*, including those encountered during spaceflight. Current investigations in our laboratory are testing this hypothesis in astronauts to explore associated changes in telomerase activity and telomere length.

As the canonical role of telomerase is to elongate telomeres, and reports of changes in telomere length following IR exposure are contradictory, suggesting both lengthening and shortening at early times postexposure, we evaluated telomere length in dividing MCF-7 and MCF-10A cells at 5 and 10 days postexposure, time points more appropriate for the assessment of surviving rather than dying cells. Interestingly, at the cell population level, telomere length was significantly shortened 5 days postexposure in both MCF-7 and MCF-10A cells. While telomere length remained significantly shorter than unirradiated controls 10 days postexposure, significant lengthening of telomeres occurred as compared to 5 days post-IR, despite both cell lines displaying significantly decreased telomerase activity during this time period. Several studies have shown association between short telomeres and radiation sensitivity (52, 55); therefore, we sought to determine whether a specific subpopulation of cells, with relatively short telomeres, was driving the response. Histogram analysis of individual telomere lengths demonstrated that all of the telomeres in the cell population were shortened and the entire distribution of telomere length was shifted monomodally to the left; thus, IR was not acting on a specific subset of radiation sensitive cells. Furthermore, the observed lengthening between days 5 and 10 also shifted the population monomodally indicating that in the cells surviving exposure, all telomeres are being lengthened, not simply preferential elongation of the critically short telomeres. These findings portend consequences for both carcinogenesis and repopulation of tumor cells following high dose radiation therapy in that short telomeres observed at 5 days could contribute to genomic instability and thus increase the propensity for carcinogenic events in surrounding normal tissue, as well as the propensity of further progression in of the tumor. Furthermore, telomere elongation in the surviving cells observed at 5–10 days postexposure strongly suggests a role in the survival/repopulation of irradiated cells and so supports blocking or manipulating this process as an effective means of preventing carcinogenesis and tumor recurrence following radiation therapy.

The suggestions that telomerase appeared to be functioning outside of its canonical role at telomeres led us to interrogate alternative possibilities for the increases in activity observed following exposure. Previous reports have suggested that telomerase activity in NCSCs and CSCs greatly exceeds that of their more differentiated counterparts, particularly with regard to mammary carcinoma. An accumulating body of evidence has begun to amass suggesting that IR induces the enrichment of CSC compartments in culture and *in vivo*. In addition, this process may be governed by the reprograming of NSCC into CSC, rather than the selection of radioresistant CSC populations, and this phenomenon may be both kinetic and highly regulated. Therefore, we speculated that the elevation of telomerase activity observed following IR exposure was the result of enriched SC populations. A time course of CD44^+^/CD24^low/−^ putative CSC populations in MCF-7 cultures following an acute 10 Gy exposure revealed a steady enrichment of CD44^+^/CD24^low/−^ cells which peaked 5 days postexposure but remained significantly elevated for at least 7 days. However, no significant enrichment of CD44^+^/CD24^low/−^ cells was observed until after 72 h, which occurred after the peak of telomerase activity had subsided. Furthermore, in MCF-10A cells, a significant, albeit transient, enrichment of CD44^+^/CD24^low/−^ cells was observed 5 days postexposure, with only slight increase in telomerase activity at early times following exposure. Thus, telomerase activity was not elevated in response to CD44^+^/CD24^low/−^ cell enrichment, but rather preceded it. CD44^+^/CD24^low/−^ dose response, clonogenic survival, and growth kinetic analysis of MCF-7 and MCF-10A cells following an acute 10 Gy exposure was conducted to further characterize the IR-induced enrichment of SC populations. The level of CSC enrichment at the peak time point of 5 days postexposure was dose dependent in MCF-7 with a significant increase detected as low as 2 Gy. In contrast, there appeared to be a threshold dose for MCF-10A of between 5 and 10 Gy for significant enrichment.

These findings led to a collaborative effort using agent-based modeling (ABM) to determine mathematically if the observed enrichment in SC compartments following IR exposure resulted from the selection of radioresistant SC populations at the time of irradiation, or conversely, was a result of reprograming non-stem into SCs. Modeling indicated that a significant reprograming component must be in place to account for the relative percentage enrichment of SC populations (in the <1% of surviving cells) 5 days postexposure, which occurred in both MCF-7 and MCF-10A. Furthermore, both reprograming and symmetric division of surviving SC populations must be employed to account for the observed enrichment (manuscript in preparation, Gao et al.).

In order to confirm that IR-induced reprograming was not unique to mammary epithelial cells, we evaluated expression of the CD34/CD38 immunophenotype in the hematopoietic cell lines KG1a, WTK1, and LCL15044. As WTK1 and LCL15044 are terminally differentiated lymphoblastoid lines that express <0.01% CD34^+^ cells at background levels, induction of reprograming was assessed using CD34 as the primary marker. In agreement with results using mammary epithelial cells, we observed a significant enrichment of CD34^+^ cells in both WTK1 and LCL15044, which increased with dose and time, out to 3 days postexposure. SC enrichment was again preceded by a general trend toward elevated telomerase activity following acute IR exposure, although neither WTK1 nor LCL15044 experienced significantly increased levels of telomerase activity. Interestingly, the only cell line that did not display IR-induced enrichment of CSC was KG1a, a therapy-induced leukemia. In this instance, the background level of CD34^+^/CD38^−^ CSCs was significantly decreased in a time- and dose-dependent manner following IR exposure. As telomerase activity was significantly increased in KG1a cells during this same time frame, the results give further credence to our claim that the elevation of telomerase activity observed following IR exposure is not an artifact of enriched SC/CSC populations. This finding is important in that it illustrates IR exposure does not serve to increase SC compartments in all situations and may in fact act differentially on existing CSC populations, perhaps especially when those populations exist at high background levels.

Finally, as increased telomerase activity did not coincide temporally with putative SC enrichment in either MCF-7 or MCF-10A cells, we hypothesized that telomerase may be playing a role to promote IR-induced SC enrichment. To test this hypothesis, we employed a small molecule inhibitor (MST-312) as well as siRNA knockdown of the catalytic hTERT component of telomerase. Both MST-312 and hTERT siRNA significantly reduced telomerase activity in both MCF-7 and MCF-10A, and both very effectively blocked IR-induced enrichment of CD44^+^/CD24^low/−^ putative SC populations evaluated 5 days post-IR exposure (10 Gy; Figure 9). These results convincingly demonstrated that telomerase activity is essential for SC enrichment in response to IR exposure and so have important implications for radiotherapy, as telomerase inhibitors are currently entering Phase III randomized trials in humans. When taken together with the observation that although elevated post-IR exposure, telomerase activity is not acting to elongate telomeres, it becomes clear that telomerase is functioning outside its canonical role. Further investigation is needed to probe underlying mechanisms, but candidate pathways include Wnt/β-catenin signaling, in which hTERT has been shown (albeit controversially) to act as a transcriptional coactivator of β-catenin target genes important for stemness and dedifferentiation (37, 39, 41, 100). Additionally, TGF-β signaling has been proposed to regulate CSC kinetics in culture and *in vivo*, and hTERT has also been implied as a downstream cotranscriptional regulator (40).

The results presented here serve to highlight and more clearly define the critical roles telomeres and telomerase play in regulating the radiation response in both normal and cancer cells. Specifically, we demonstrated that loss of telomere end-capping function results in increased mutation burden and chromosomal events that fuel instability; furthermore, this mutation burden may be especially elevated in response to high LET radiation, particularly in the context of POT1 deficiency. We also establish that telomerase is activated by IR exposure, but the extent of such elevation is dose, dose rate, and cell type dependent, making assessment of risks posed by IR-induced increases in telomerase activity complex and requiring further exploration. Third, we confirmed and elaborated upon previous findings that acute low LET IR exposure enriches putative mammary CSCs in culture and expanded these studies to include lymphoblastoid lines, which are of great relevance to carcinogenesis and spaceflight risk. Lastly, we demonstrated the requirement of telomerase activity in promoting IR-induced putative SC enrichment in mammary epithelial cells of both cancer and non-cancer origin, a finding with important implications for radiation therapy. Taken together, these findings serve to strengthen the view that telomeres and telomerase are far more than casual observers constrained to the ends of chromosomes. Rather, they occupy a central role in the cellular radiobiological response, governing everything from cellular lifespan (aging), cellular plasticity (SCs), and genomic integrity (instability), to survivability and carcinogenic potential following exposure.

## Author Contributions

BS made substantial contributions to the concept and design of the work, as well as the acquisition, analysis and interpretation of the data, drafting, and critically revising the work. CN, MM, CB, AH, and RI contributed to the acquisition and analysis of data for the work, and critically revising. HL and SB made substantial contributions to the concept and design of the work and to drafting and revising the work. All authors gave approval and agree to be accountable for all aspects of the work.

## Conflict of Interest Statement

The authors declare that the research was conducted in the absence of any commercial or financial relationships that could be construed as a potential conflict of interest.

## Supplementary Material

Figure S1 **Western blot validation of siRNA knockdown of telomeric binding proteins TRF1, TRF2, and POT1 in WTK1 cells**.

Figure S2 **Representative scatter plots of MCF-7 and MCF-10A cells. (A) Immunotyped based on the expression of cell surface markers CD44 and CD24 and (B) validation of stem cell-like properties using aldefluor assay**.
