# Supplementary figures and images for: Telomeres and Telomerase in the Radiation Response: Implications for Instability, Reprograming, and Carcinogenesis
Source: Front Oncol. 2015 Nov 24;5:257. doi: 10.3389/fonc.2015.00257 (PMC4656829; doi:10.3389/fonc.2015.00257)

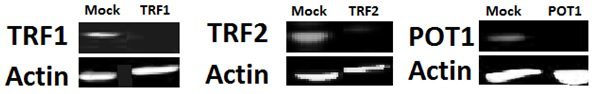

Supplement: Figure S1 — Western blot validation of siRNA knockdown of telomeric binding proteins TRF1, TRF2, and POT1 in WTK1 cells. [file Image_1.JPEG]

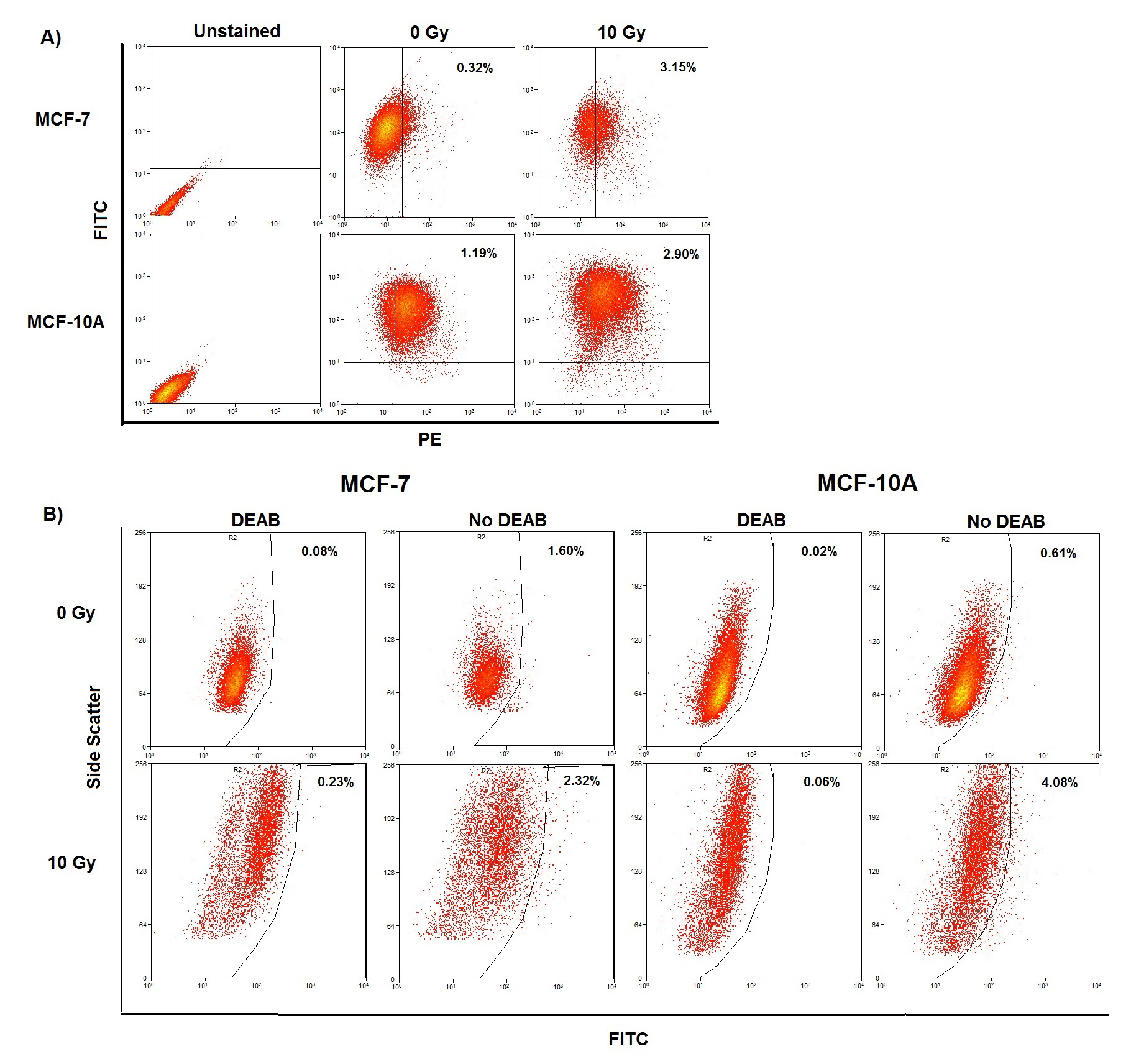

Supplement: Figure S2 — Representative scatter plots of MCF-7 and MCF-10A cells. (A) Immunotyped based on the expression of cell surface markers CD44 and CD24 and (B) validation of stem cell-like properties using aldefluor assay. [file Image_2.JPEG]
